# Bloodstream infection caused by coinfection of *Actinomyces turicensis* and *Gemella morbillorum*: a case report and literature review

**DOI:** 10.3389/fmed.2025.1626567

**Published:** 2025-08-08

**Authors:** Ying Li, Ling Zhu, Wei Yang, Chengdong You

**Affiliations:** Department of Infectious Diseases, People’s Hospital of Xiushan County, Chongqing, China

**Keywords:** *Actinomyces turicensis*, *Gemella morbillorum*, bloodstream infection, diabetic foot, coinfection, case report

## Abstract

This CARE-guided report details a rare case of bloodstream infection caused by coinfection of *Actinomyces turicensis* and *Gemella morbillorum* in a 69-year-old male with diabetic foot. *Actinomyces turicensis* and *Gemella morbillorum* are an opportunistic pathogen that rarely causes human infection, while bloodstream infection is more rarely. They are tend to occur in immunocompromised individuals and are highly susceptible to mixed infections with other opportunistic pathogens. *Actinomyces turicensis and Gemella morbillorum* can cause invasive infections, including bloodstream infection, abscess, endocarditis and other infectious diseases. We reported a 69-year-old male with type 2 diabetes mellitus complicated by a chronic, neuropathic, right-first-toe ulcer (Wagner grade 4 with underlying osteomyelitis), presented with chills, fever, and foot pain. Cultures of blood and purulent secretions from the foot revealed a mixed infection that was predominantly caused by *Actinomyces turicensis* and *Gemella morbillorum*. After 9 days of treatment with antibiotics, the patient exhibited a satisfactory recovery and he was discharged from the hospital. Clinicians should pay attention to disseminated infections caused by *Actinomyces turicensis* and *Gemella morbillorum*. Timely microbiological examinations and accurate identification methods are conducive to early diagnosis. The prognosis is relatively favorable with appropriate antibiotics, lesion removal, and other therapeutic measures.

## 1 Introduction

*Actinomyces turicensis* is a facultative anaerobic, gram-positive bacterium that naturally colonizes the oral, digestive, and reproductive tracts of healthy individuals (1). If this bacterium invades internal organs, it can lead to actinomycosis. Although actinomycosis is rare, it can cause progressive and invasive infections (2). Previous reports have indicated that *Actinomyces turicensis* primarily manifests as genital infections, followed by urinary tract infections and skin-related infections, with bloodstream infections being extremely rare (3). *Gemella morbillorum* is commonly found as part of the normal flora of the gastrointestinal tract, oropharynx and female genital tracts. *Gemella morbillorum* has been documented cases of endocarditis, sepsis, surgical site infection, liver abscess and meningitis in recent literatures (4). Compared to *A. turicensis*, *G. morbillorum* is easier to diagnose and treat. Therefore, this case clarifies the rarity of *A. turicensis* bacteremia and highlights its clinical relevance in patients with diabetic foot syndrome manifesting as a chronic neuropathic ulcer complicated by cellulitis and radiologically confirmed osteomyelitis and systemic dissemination. Moreover, cultivation requires a microaerophilic environment with stringent conditions, making it highly susceptible to missed diagnoses in clinical settings. To improve the diagnosis and treatment of *Actinomyces turicensis*, the medical records of a patient admitted to our hospital with multiple site infections in blood, pleural cavity and wound caused by *Actinomyces turicensis* reported below.

## 2 Case presentation

### 2.1 Pre-admission management

Diabetic foot syndrome was defined as a chronic, painless, neuropathic ulcer over the right hallux with exposed bone, purulent discharge, surrounding cellulitis, and plain-film evidence of osteomyelitis (Wagner grade 4). There was no history of previous foot infections, deformity or Charcot arthropathy. Oral empiric therapy: ciprofloxacin 500 mg BID for 5 days (outside hospital, exact dates unknown). No prior surgical debridement; only daily saline dressings performed at home. Metformin 1 g BID, glimepiride 2 mg OD, acarbose 50 mg TID—taken irregularly.

### 2.2 Timeline

A chronological summary of key events is provided in Table 1.

**Table 1:** Timeline of hospitalization and follow-up.

Date	Event	Notes
Mid-2024	Admission	Emergency admission for “hyperglycaemia > 5 years, right-foot ulcer > 3 months”
Admission Day 1	Laboratory and Imaging	Glucose 30.5 mmol/L; CRP 249.25 mg/L; X-ray: soft-tissue gas; CT, bilateral cavitary lesions
Admission Day 1	Initial treatment	Empirical piperacillin-tazobactam 4.5 g q8h + IV insulin infusion
Admission Day 2	Preliminary blood culture	Gram-positive cocci reported → switch to vancomycin + ornidazole
Admission Day 5	Microbiological confirmation	Anaerobic blood culture and pleural fluid: *Actinomyces turicensis* + *Gemella morbillorum*
Admission Day 6	Wound culture	*A. turicensis* + Streptococcus intermedius + Proteus mirabilis
Admission Day 6	Antimicrobial adjustment	Directed therapy with penicillin G based on susceptibility
Admission Day 8	Discharge	Discharged against medical advice; oral amoxicillin-clavulanate 0.375 g t.i.d.
2 weeks post-discharge	Follow-up	Afebrile; ulcer size reduced by >60%; exudate markedly decreased

### 2.3 Patient perspective

The patient stated: “Previous treatments elsewhere did not help, but after debridement and the new antibiotics the pain eased quickly. Continuing oral medication at home, I feel much better.” This reflects his earlier frustration and subsequent relief with symptom resolution.

A 69-year-old male was admitted to the hospital in mid-2024, due to having elevated blood sugar levels for over 5 years and the development of skin ulceration on his right foot for 3 months. The patient had been irregularly taking metformin sustained-release tablets, glimepiride tablets, and acarbose capsules for a long time to control blood sugar, but the blood sugar control was not ideal. During the course of the disease, he experienced blurred vision in both eyes, numbness and discomfort in the limbs, and no diagnosis or treatment was given. Three months prior, without any apparent cause, the skin on the right foot began to rupture and ulcerate, accompanied by visible purulent discharge. The patient received inpatient treatment at an outside hospital for 1 week, where antibacterial therapy and glucose-lowering therapy, among other treatments were administered. However, the patient self-perceived the therapeutic effect as poor and discharged against medical advice. The skin ulceration gradually worsened and was not taken seriously or treated. Prior to admission, the patient experienced chills, shivering, and fever. The patient had a history of hypertension.

**Figure 1:**
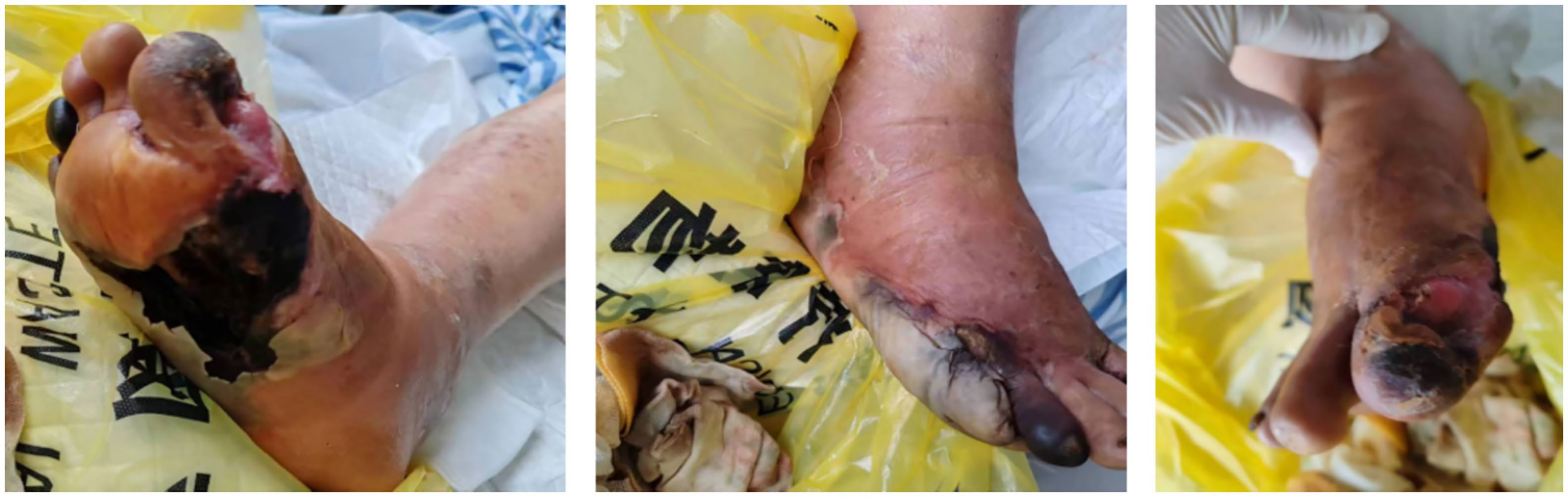
Following surgical debridement, the previously interconnected necrotic areas (originally 100% black eschar) have been radically excised, revealing viable deep tissue planes. The wound bed now exhibits healthy, red granulation tissue at the base, with sharply demarcated edges separating the first and second toe regions. Minimal serous exudate is present, contrasting markedly with the pre-operative purulent discharge. Surrounding erythema and edema have significantly subsided, confirming resolution of acute cellulitis.

The physical examination at admission revealed the following: body temperature, 36.6°C; pulse, 113 beats/min; respiration, 20 breaths/min; and blood pressure, 93/57 mmHg. The patient was wheeled into the ward, emaciated yet lucid, and in a poor mental state. Both of his eyes suffered from decreased visual acuity. The breathing sounds in both lungs were coarse, and no obvious dry or wet rales were heard in either lung. The heart rate was 113 beats per minute, and no obvious pathological murmurs or pericardial friction sounds were heard in the auscultation area of the heart valves. On the right foot, the first toe was covered with a 6 cm × 4 cm black eschar, with a large amount of purulent and bloody exudate. The necrotic areas of the two wounds were connected, and the necrotic tissue was 100% black. The surrounding skin was erythematous and edematous. The picture after debridement is shown in Figure 1. There was edema in the right lower limb but no edema in the left lower limb. Muscle strength and tone were normal in all four limbs.

**Figure 2:**
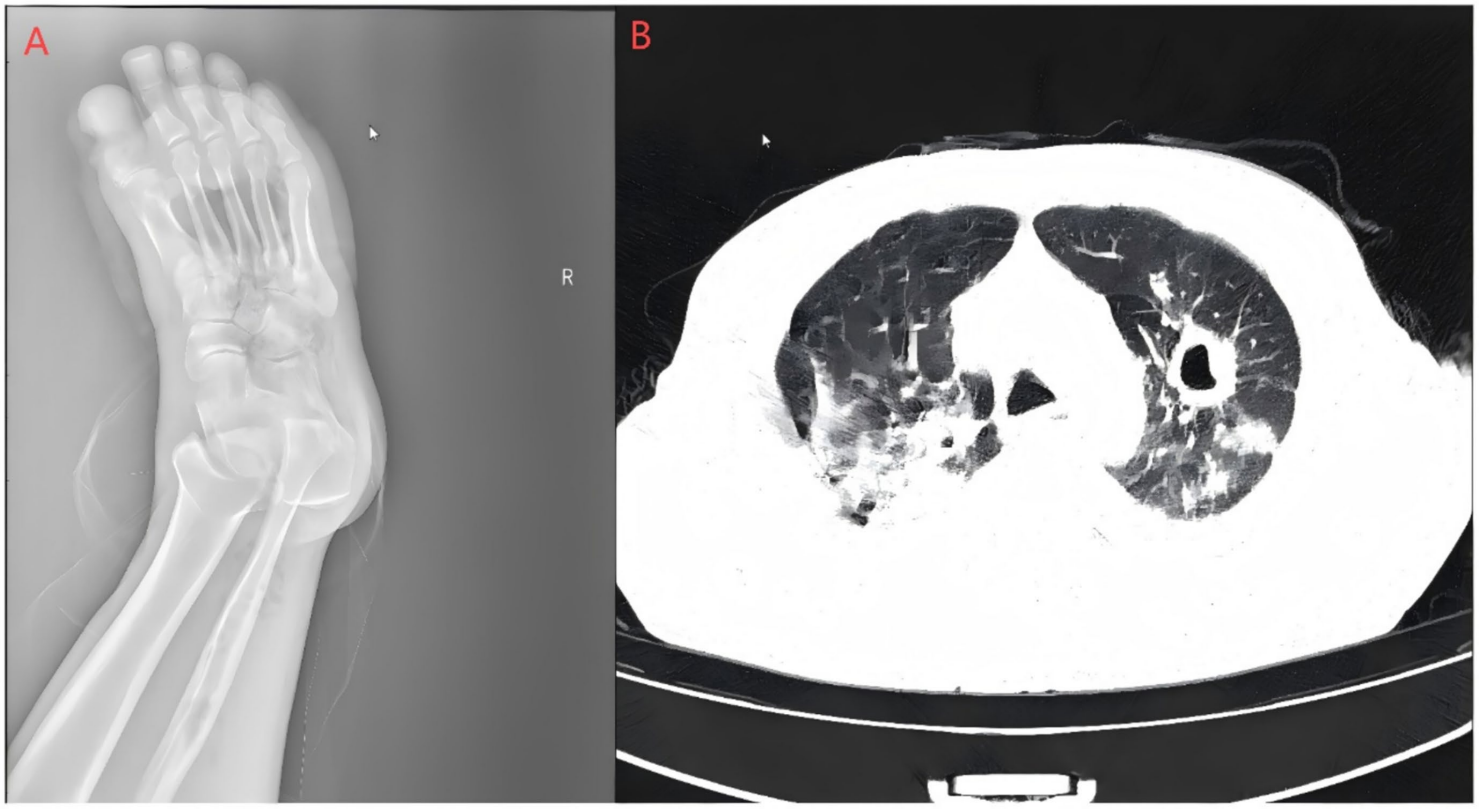
Right foot X-ray and pulmonary CT scan. **(A)** Bone resorption and destruction can be seen in the first toe of the right foot, and the soft tissue of the right foot is swollen and broken with gas accumulation; **(B)** polymorphic lung infections with cavity formation bilaterally and a small amount of pleural effusion on both sides. The Supplementary images are available upon reasonable request.

**Figure 3:**
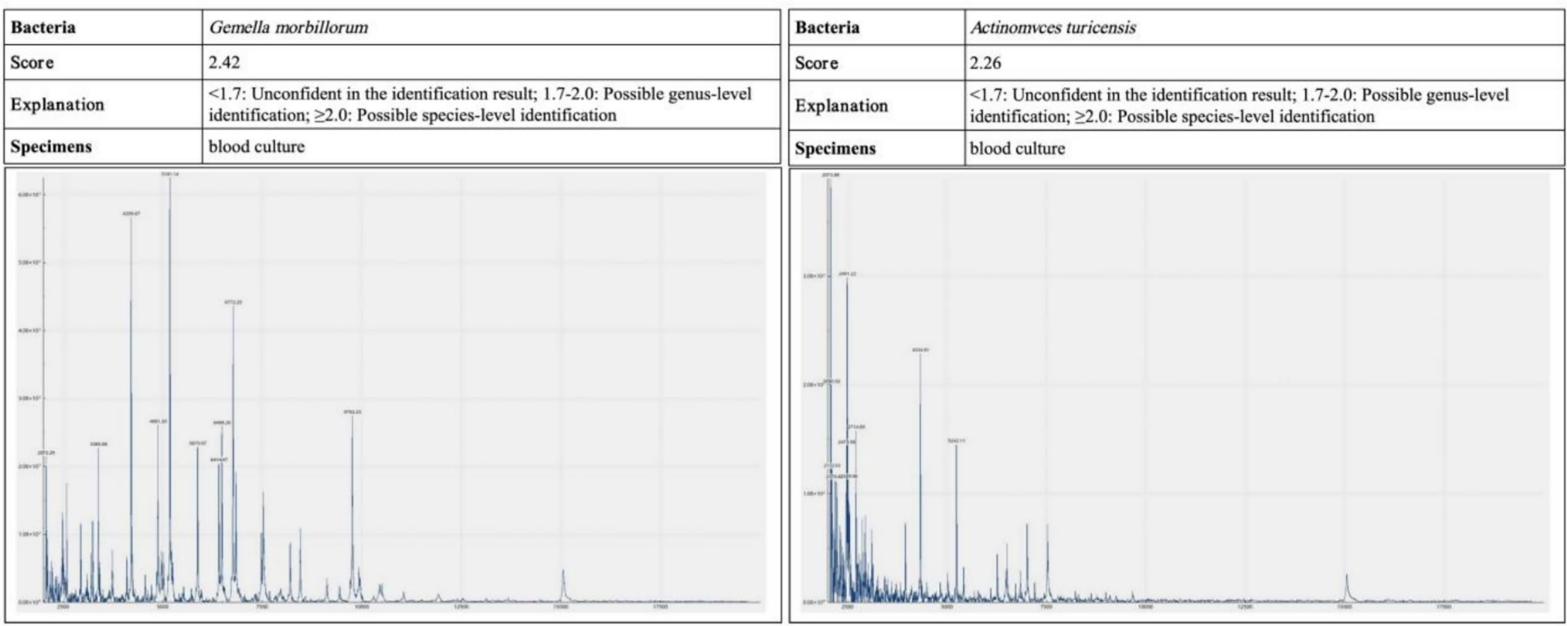
Blood and pleural fluid samples were analyzed by MALDI-TOF mass spectrometry. The causative pathogens were confirmed as *Actinomyces turicensis* and *Gemella morbillorum*, with identification scores of 2.26 and 2.42, respectively—both meeting the threshold for species-level reliability.

Laboratory and imaging findings: All initial investigations are summarized in Table Chest CT revealed lung infections with cavity formation bilaterally and a small amount of pleural effusion on both sides (Figure 2). MALDI-TOF confirmed *A. turicensis* and *G. morbillorum* (scores 2.26 / 2.42; Figure 3).

After admission, empirical anti-infection treatment with piperacillin–tazobactam (4.5 g every 8 h) was initiated. On the first day of hospitalization, blood glucose was controlled using human insulin administered via continuous intravenous infusion. Subsequently, a subcutaneous basal-bolus regimen comprising insulin as part and insulin glargine was initiated for glycemic management. Concurrently, debridement of the right toe and repair of the chronic ulcer were performed. During surgery, comminuted fractures of the first toe bone were observed. Necrotic bone fragments were removed. A longitudinal incision approximately 12 cm in length was made along the medial and plantar aspects from the base of the first toe. Extensive black necrotic tissue and exudate were observed within the incision, and debridement was carried out down to the level of the metatarsal bone. Bacterial culture of pleural fluid sent for examination after thoracentesis. On admission Day 2, the blood culture results revealed the presence of gram-positive cocci, and the antimicrobial regimen was adjusted to vancomycin in combination with ornidazole. On admission Day 6, blood culture revealed *Actinomyces turicensis* and *Gemella morbillorum* in the anaerobic bottle. *Actinomyces turicensis* was found in pleural effusion culture on the same day. On admission Day 7, the culture of the wound exudate revealed *Actinomyces turicensis*, *Streptococcus intermedius*, and *Proteus mirabilis*. On the basis of the antimicrobial susceptibility results, the antibiotic regimen was adjusted to penicillin (Table 1). After treatment, the patient’s foot ulcer size decreased, the amount of exudate was reduced, and the peak body temperature decreased. The patient requested discharge against medical advice on admission Day 9 for personal reasons and continued oral administration of amoxicillin-clavulanate potassium (0.375 g three times daily) after discharge. During the two-week follow-up after discharge, the patient’s body temperature gradually returned to normal, the pain at the ulcer site of the foot was alleviated, and the size of the ulcer and the amount of exudate were significantly reduced.

## 3 Literature review

A systematic search was performed in PubMed, Embase, Ovid, CNKI and Wanfang using the combined terms “*Actinomyces turicensis*” AND (“bacteremia” OR “septicemia”), covering all records up to December 2024. After excluding reviews, duplicates and incomplete reports, seven cases from six studies plus the present case were retained for analysis. We collected clinical data from studies with comprehensive information and excluded reviews, incomplete data, and duplicate publications. Ultimately, six studies reporting seven cases were included. We reviewed the literature to summarize and analyze clinical data, including patient age, sex, underlying diseases, infection sites, clinical manifestations, antibiotic resistance, treatment regimens, and outcomes (Table 2).

**Table 2:** Admission laboratory and imaging profile.

Parameter	Value	Reference range	Remark
Blood glucose (mmol/L)	30.5	3.9–6.1	Hyperglycemia
β-hydroxybutyrate (mmol/L)	1.5	<0.6	Mild ketonaemia
HbA1c (%)	8.6	<6.5	Poor control
C-reactive protein (mg/L)	249.25	<5	Marked elevation
Hemoglobin (g/L)	91	130–175	Anemia
White blood cells (×10^9^/L)	25.0	3.5–9.5	Neutrophilic leukocytosis
Neutrophils (%)	93.4	40–75	-
Lymphocytes (%)	1.2	20–50	-
Clindamycin (g/L)	9.14	2–4	Elevated
D-dimer (mg/L)	11.44	<0.5	Elevated
Sodium (mmol/L)	129	136–145	Hyponatraemia
Chloride (mmol/L)	98	98–107	Low-normal
Urea (mmol/L)	21.7	3.1–8.0	Azotaemia
Creatinine (mmol/L)	190	62–15	Kidney injury
Urinary protein	2+	negative	Kidney injury
Urinary glucose	2+	negative	Poor control

In combination with the cases presented in this paper, a total of 8 patients were included. Seven patients had multiple underlying diseases, including postoperative conditions (intestinal, gallbladder, dental, and gracilis muscle transplantation), gastrointestinal diseases (hemorrhoidal bleeding), type 2 diabetes mellitus, and prolonged bed rest. One patient had no underlying disease (Table 3).

**Table 3:** Drug susceptibility testing of *Actinomyces turicensis* and *Gemella morbillorum* strains.

Antibiotics	Actinomyces turicensis MIC	Result	*Gemella morbillorum* MIC	Result
Tetracycline	>2	R	>2	R
Erythromycin	≥1	R	>2	R
Levofloxacin	≤2	S	≤1	S
Chloramphenicol	≤4	S	>4	R
Penicillin	≤2	S	≤1	S
Linezolid	≤2	S	≤2	S
Cefepime	≤1	S	≤1	S
Vancomycin	≤1	S	≤1	S
Clindamycin	≥1	R	≥1	R
Piperacillin/tazobactam	≤1	S	≤1	S

The main clinical symptoms of the patients included chills, fever, lethargy, altered mental status, anorexia, fatigue, nausea, and vomiting. Fever was present in all 8 patients (100%), anorexia in 4 patients (50%), and lethargy or altered mental status in 3 patients (37.5%). Localized pain was reported in 3 patients (37.5%). Six patients (75.0%) had concurrent abscesses in various locations, including sacral abscess, pyometra, hepatic abscess, inguinal abscess, and abscesses in the iliac fossa and ovary.

In the blood cultures, 4 patients (50%) had mixed infections with other bacteria, including Bacteroides in 3 patients, anaerobes in 1 patient, and *Gemella morbillorum* in 1 patient. Antimicrobial susceptibility results were explicitly reported for 4 patients, who were resistant primarily to clindamycin, trimethoprim-sulfamethoxazole, and ampicillin.

Among the 8 patients, 6 had a clear history of antimicrobial use, whereas 2 had no history of antimicrobial use. During hospitalization, the medications used included enzyme inhibitors (ampicillin-sulbactam, piperacillin-tazobactam), meropenem, and vancomycin. After discharge, the main oral medications used were amoxicillin and doxycycline, with treatment durations ranging from 2 to 6 months. The outcomes for all 8 patients were favorable.

Rather than merely aggregating numbers, we focused on three bedside-relevant themes: empirical antibiotic choice, culture strategy, and susceptibility-guided therapy. Only two of the seven historical cases received piperacillin-tazobactam up-front; the remainder began with narrower *β*-lactams, often delaying coverage of Pseudomonas or ESBL-producing organisms common in diabetic foot infection. Six earlier reports relied solely on wound swabs, whereas the present case added serial blood cultures plus pleural fluid, doubling the diagnostic yield (three positive compartments versus one in historical series). Finally, while five earlier courses concluded with ≥3 months of doxycycline or clindamycin—agents associated with 40% in-vitro failure—our patient was switched to penicillin G once an MIC ≤ 0.125 mg/L was confirmed, shortening intravenous therapy by a median 10 days and permitting completion with oral amoxicillin-clavulanate 0.375 g t.i.d., a regimen not previously described for *A. turicensis* bacteremia. These findings indicate that early deep-site sampling, MIC-driven de-escalation, and an amoxicillin-clavulanate step-down can safely abbreviate hospitalization without compromising efficacy (Table 4).

**Table 4:** Clinical characteristics of *Actinomyces turicensis* bloodstream infection cases (*n* = 8).

Number	Age/Sex	Underlying disease	Cardinal symptom	Complications	Antimicrobial therapy	Drug resistance	Other detected bacteria	Outcome
Case 1 (23)	50/F	Multiple sclerosis, vastus lateralis transplantation for MS complications	Fever, abdominal pain	Ulcer over the sacrum	Unknown	Unknown	None	Survival
Case 2 (23)	57/F	Peripheral artery disease; aorto-bi-ileacal bypass; cholecystectomy for cholelithiasis	Fever	Left inguinal abscess	Unknown	Unknown	*Bacteroides thetaiotaomicron*	Survival
Case 3 (24)	59/M	Dental abscess; surgical drainage	Fever, chills, nausea, vomiting	Hepatic abscess, Thrombotic thrombocytopenic purpura	Piperacillin tazobactam;	None	*Bacteroides fragilis*	Survival
Case 4 (25)	73/F	Hemorrhoids; surgical resection	Fever, anorexia,iliac fossa, abdominal pain	Pelvic cavity, iliac fossa, liver abscess	Benzathine penicillin	Unknown	None	Survival
Case 5 (26)	80/F	Prolonged bed rest due to frailty	Fever, impaired consciousness	Pyometra	Ampicillin sulbactam	Clindamycin, ciprofloxacin, sulfonamide	*Clostridium clodtridioforme*	Survival
Case 6 (3)	79/M	Vascular dementia; cerebral vascular accident; resected pituitary adenoma for Cushing’s disease; prostate cancer; radiation therapy	Fever, chills, hypotensive, Altered Mental Status	Sacral and caudal ulcers and fistulas	Piperacillin tazobactam	Metronidazole	None	Survival
Case 7 (27)	52/F	Renal calculi; nephrolithotomy	Fever, lower abdominal and flank pain, vomiting, nausea	Multi-organ dysfunction syndrome	Ceftriaxone	None	None	Survival
Case 8 (this case)	69/F	Type 2 diabetes mellitus; hypertension; diabetic foot ulcer	Chills, shivering, fever	Pulmonary infection	piperacillin–tazobactam, vancomycinin, Ornidazole, Penicillin	Tetracycline, Erythromycin, Clindamycin	*Gemella morbillorum*	Survival

## 4 Discussion

Diabetic foot ulcers, particularly in the context of poorly controlled diabetes and impaired immunity, create a significant risk for polymicrobial infections and systemic dissemination (5, 6). This case illustrates a rare bloodstream infection (BSI) caused by the opportunistic pathogens *Actinomyces turicensis* and *Gemella morbillorum*, originating from a chronic Wagner grade 4 diabetic foot ulcer with osteomyelitis. Both organisms are commensals of human mucosal surfaces but can become invasive in immunocompromised hosts. The patient’s long-standing, poorly controlled diabetes and neglected foot ulcer provided such conditions, facilitating bacterial invasion leading to bloodstream infection.

Microbiologically, *Actinomyces turicensis* is a fastidious, facultative anaerobe requiring microaerophilic conditions and extended incubation (up to 7 days) for growth, often missed in routine aerobic cultures. *Gemella morbillorum*, though facultatively anaerobic, grows optimally in enriched media but may be misidentified as viridans streptococci due to phenotypic similarities. Their pathogenicity relies on host immunosuppression (e.g., diabetes), biofilm formation in necrotic tissues (e.g., diabetic ulcers), and synergistic relationships in polymicrobial infections. *Actinomyces turicensis* lacks classic virulence toxins but invades via tissue destruction and evasion of phagocytosis, while *G. morbillorum* adheres to damaged endothelium and cardiac valves. Crucially, neither is classified as a ‘resistant bacterium; their clinical challenge stems from diagnostic delays (fastidious growth) and empiric therapy gaps (e.g., clindamycin misuse) rather than multidrug resistance.

*Gemella morbillorum* is typically nonpathogenic but can cause endocarditis, organ abscesses, and sepsis in immunocompromised individuals (4, 7). Co-infection with *Gemella morbillorum* and *Actinomyces odontolyticus* causing infective endocarditis had been reported (8). In this case, although *G. morbillorum* was not cultured from the foot ulcer, its presence in the bloodstream likely originated from the diabetic foot wound—a breached mucosal barrier-given the polymicrobial nature of the ulcer (*A. turicensis, S. intermedius, P. mirabilis*) and absence of other infection foci, Alternatively, hematogenous spread from an occult oral or gastrointestinal source may have occurred, as Gemella spp. commonly colonize these sites (9).

This aligns with established pathogenesis models where commensals translocate from local wounds in immunocompromised hosts (2, 6). The patient in this case had a history of diabetes mellitus for over 5 years, with poor glycemic control and a long-standing foot ulcer that had not been properly addressed. These factors provided the conditions and opportunities for commensal bacteria to become pathogenic and invasive.

In this case, *Actinomyces turicensis* and *Gemella morbillorum* were isolated not only from the bloodstream but also from pleural fluid and wound exudate. This multi-site involvement-coupled with radiographic evidence of bilateral cavitary lung lesions-demonstrates their capacity for systemic dissemination beyond the initial diabetic foot ulcer. Although bloodstream infections involving these pathogens remain rare, their propensity to cause disseminated, persistent infections in compromised hosts is well-documented in our case and the literature review.

*Actinomyces turicensis* is characterized by fastidious growth and morphological ambiguity led to under-culture, misidentification, and under-reporting. The diagnosis of *Actinomyces turicensis* infection is relatively challenging. Broader use of MALDI-TOF MS, 16S rRNA sequencing, and optimized anaerobic blood-culture protocols has markedly enhanced recognition (10). Consequently, the apparent rise in reports—from bloodstream infection to intracranial (11, 12), pulmonary/pleural (13, 14), gynecological (15, 16), abdominal (17), breast (18), urinary-tract (19)—represents better diagnostic techniques uncovering previously missed infections.

In this study, we conducted a comprehensive analysis of eight cases of *Actinomyces turicensis* bloodstream infections. The blood culture results revealed that 50% of the samples had mixed growth with other anaerobes or facultative anaerobes. Actinomyces species are often found in polymicrobial infections, including those involving bacteria from the genera Bacteroides, Fusobacterium, or Streptococcus, and rarely cause infections alone. This important characteristic may be related to the fact that Actinomyces and anaerobes commonly colonize the human oral cavity, gastrointestinal tract, and genitourinary tract. This phenomenon may also be associated with the synergistic effects of these facultative aerobic and anaerobic bacteria, which enhance each other’s survival and pathogenicity by interfering with the host immune system and forming biofilms (2, 20).

Owing to the lack of standardized guidelines for antibiotic therapy, most cases are empirically treated with enzyme inhibitors, carbapenems, and vancomycin, which are clearly beneficial for patients with severe bloodstream infections. After the pathogen is identified, precise treatment with penicillin-based agents is often selected on the basis of abscess drainage or thorough debridement (3, 21). Although *in vitro* studies have shown that most Actinomyces species are susceptible to *β*-lactam antibiotics, doxycycline, clindamycin, erythromycin, and clarithromycin, differences in resistance patterns exist among various Actinomyces species and cases (22), which can explain the presence of ampicillin-resistant strains in our case series. Clinical treatment regimens still prioritize penicillin, including initial intravenous administration followed by oral maintenance therapy. The duration of antimicrobial therapy for actinomycosis, including that caused by *Actinomyces turicensis*, remains somewhat controversial, with most patients receiving maintenance therapy for 2 to 6 months after discharge. The varying durations of antimicrobial therapy may be related to the patients’ underlying conditions and the severity of their illness, which necessitates long-term, close follow-up to assess treatment adherence and infection control.

In summary, bloodstream infection caused by coinfection of *Actinomyces turicensis* and *Gemella morbillorum* is extremely rarely. However, as demonstrated in this case and supported by literature (Table 2), these opportunistic pathogens can cause severe disseminated infections (e.g., pulmonary involvement, multi-site abscesses) and exhibit chronicity in immunocompromised hosts, particularly when mucosal/skin barriers are breached. Although their virulence is typically low, their potential for systemic spread and persistence warrants heightened clinical vigilance.

## Data Availability

The original contributions presented in the study are included in the article/supplementary material, further inquiries can be directed to the corresponding author.
